# The gut microbiota–bile acid axis: A potential therapeutic target for liver fibrosis

**DOI:** 10.3389/fcimb.2022.945368

**Published:** 2022-09-15

**Authors:** Yu-Lin Zhang, Zhen-Jiao Li, Hong-Zhong Gou, Xiao-Jing Song, Lei Zhang

**Affiliations:** ^1^ The First Clinical Medical College, Lanzhou University, Lanzhou, China; ^2^ Department of General Surgery, The First Hospital of Lanzhou University, Lanzhou, China; ^3^ Key Laboratory of Biotherapy and Regenerative Medicine of Gansu Province, The First Hospital of Lanzhou University, Lanzhou, China

**Keywords:** gut microbiota, bile acid, gut microbiota–bile acid axis, liver fibrosis, chronic liver disease

## Abstract

Liver fibrosis involves the proliferation and deposition of extracellular matrix on liver tissues owing to various etiologies (including viral, alcohol, immune, and metabolic factors), ultimately leading to structural and functional abnormalities in the liver. If not effectively treated, liver fibrosis, a pivotal stage in the path to chronic liver disease, can progress to cirrhosis and eventually liver cancer; unfortunately, no specific clinical treatment for liver fibrosis has been established to date. In liver fibrosis cases, both the gut microbiota and bile acid metabolism are disrupted. As metabolites of the gut microbiota, bile acids have been linked to the progression of liver fibrosis *via* various pathways, thus implying that the gut microbiota–bile acid axis might play a critical role in the progression of liver fibrosis and could be a target for its reversal. Therefore, in this review, we examined the involvement of the gut microbiota–bile acid axis in liver fibrosis progression to the end of discovering new targets for the prevention, diagnosis, and therapy of chronic liver diseases, including liver fibrosis.

## 1 Introduction

Liver fibrosis, which is a pathological repair response to various injuries in the liver, is a common stage in the progression of chronic liver disease to cirrhosis (Campana and Iredale, 2017). Its pathogenesis is primarily characterized by intrahepatic fibrous hyperplasia due to the abnormal distribution and excessive deposition of extracellular matrix material after the activation of hepatic myofibroblasts, such as hepatic stellate cells (Parola and Pinzani, 2019). Studies have shown that liver fibrosis is a potentially reversible dynamic process that can progress to irreversible cirrhosis if the inducers of fibrosis are not eliminated; thus, the early reversal of liver fibrosis is critical (Povero et al., 2010). However, to date, no clinically effective therapy for this disease has been established (Roehlen et al., 2020).

Additionally, the gut microbiota of patients with liver fibrosis is considerably altered, possibly having a significant effect on the pathogenesis of the disease (De Minicis et al., 2014; Boursier et al., 2016; Xie et al., 2021). Bile acids, which are the metabolites of the gut microbiota, are disrupted in patients with liver fibrosis. The disrupted bile acids can alter the composition of the gut microbiota and can regulate liver fibrosis *via* several pathways (Caussy et al., 2019; Keitel et al., 2019; Liu et al., 2019b; Adams et al., 2020; Chen et al., 2020a; Funabashi et al., 2020; Wang et al., 2020; Nimer et al., 2021; Xie et al., 2021). These previous studies suggest that gut microbiota can influence liver fibrosis by affecting bile acid metabolism and signaling processes. Therefore, in this review, we evaluated the role of the gut microbiota–bile acid axis in liver fibrosis progression and discussed several potential treatments for liver fibrosis that target the said axis.

## 2 Gut microbiota and liver fibrosis

Gut microbiota, which constitutes the microbiota present in the intestine, plays an important role in the nutritional, immune, and biological barrier functions of the body (Marchesi and Ravel, 2015; Ramírez-Pérez et al., 2017; Barko et al., 2018; Zhang et al., 2018). Specifically, normal human gut microbiota predominantly consists of Firmicutes, Bacteroidetes, Proteobacteria, Actinobacteria, Fusobacteria, and Verrucomicrobia, with Firmicutes and Bacteroidetes accounting for more than 90% (Eckburg et al., 2005; Lozupone et al., 2012). However, numerous studies have shown that the gut microbiota of patients with liver fibrosis is disturbed and that this disruption plays an important role in liver fibrosis progression (Chen et al., 2011; Bajaj et al., 2012b; Kakiyama et al., 2013; Bajaj et al., 2014; Kakiyama et al., 2014; Bajaj et al., 2015; Boursier et al., 2016; Lelouvier et al., 2016).

### 2.1 Alteration of gut microbiota in patients with liver fibrosis

The gut microbiota of patients with liver fibrosis is significantly altered, with a decrease in the diversity and richness, an increase in potentially pathogenic bacteria, and a decrease in beneficial bacteria (Bajaj et al., 2012a; Kakiyama et al., 2013). Most studies have used the microbiota of stool samples to characterize the state of the body’s gut microbiota. Moreover, some studies have revealed changes in the microbiota of the duodenal mucosa, sigmoid mucosa, blood, and saliva from patients with liver fibrosis (Bajaj et al., 2012a; Bajaj et al., 2015; Chen et al., 2016; Lelouvier et al., 2016) (**Table 1**).

**Table 1:** Alteration of gut microbiota in patients with liver fibrosis.

Causes of fibrosis/type of liver disease	Groups compared	Methodology	Results		References
			Increased	Reduced	
NAFLD	Minimal or no fibrosis vs. advanced fibrosis	16S rRNA gene sequencing Stool sample	*Prevotella copri, Lachnobacterium, Ruminococccaceae*	*Bacteroides*	(Dong et al., 2020)
NAFLD	Non-severe (fibrosis < 2) vs. severe (fibrosis 2+) fibrosis	16S rRNA gene sequencing, Metagenomic shotgun sequencing Stool sample	*Bacteroidetes, Proteobacteria*, Enterococcaeae *TM7, Lactobacillus*	*Fusobacteria, Verrucomicrobia, Firmicutes, Akkermansia*	(Schwimmer et al., 2019)
NAFLD	Mild/moderate (stage 0–2 fibrosis) vs. advanced fibrosis (stage 3/4 fibrosis)	Metagenomic shotgun sequencing Stool sample	*Proteobacteria, Bacteroides vulgatus, Escherichia coli*	*Firmicutes, Eubacterium rectale, Ruminococcus obeum CAG: 39, R. obeum*	(Loomba et al., 2017)
NAFLD	No Fibrosis vs. fibrosis	16S rRNA gene sequencing Stool sample	*Proteobacteria, Bacteroidetes, Fusobacteria, Fusobacteriaceae*	*Firmicutes, Actinobacteria, Ruminococcaceae, Lachnospiraceae, Coriobacteriaceae*	(Lelouvier et al., 2016)
		16S rRNA gene sequencing Blood sample	*Proteobacteria*, *Sphingomonas, Bosea*	*Actinobacteria, Variovorax*
NAFLD	F0/1 fibrosis vs. F ≥ 2 fibrosis	16S rRNA gene sequencing Stool sample	*Bacteroidaceae, Bacteroides, Ruminococcus*	*Prevotellaceae, Erysipelotrichaceae, Prevotella*	(Boursier et al., 2016)
HBV-induced cirrhosis, PBC	Controls vs. cirrhosis	16S rRNA gene sequencing Duodenal mucosal sample	*Veillonella, Megasphaera, Dialister, Atopobium, Prevotella*	*Neisseria, Haemophilus, SR1 genera incertae sedis*	(Chen et al., 2016)
HCV-induced cirrhosis, ALC, NASH	Controls vs. cirrhosis	Multi-tagged pyrosequencing Saliva sample	*Enterobacteriaceae* *Enterococcaceae*	(*Lachnospiraceae + Ruminococcaceae + Clostridiales Incertae Sedis XIV/Streptococcaceae*)	(Bajaj et al., 2015)
Non-ALC, ALC	Non-ALC vs. ALC	Multi-tagged pyrosequencing Stool sample	*Bacterioidaceae*	*Veillonellaceae*	(Kakiyama et al., 2014)
Hepatitis B, C, D, E-induced cirrhosis, ALC, PBC, schistosomiasis cirrhosis, autoimmune cirrhosis	Controls vs. cirrhosis	Metagenomic shotgun sequencing Stool sample	*Proteobacteria, Fusobacteria, Veillonella, Streptococcus, Clostridium, Prevotella*	*Bacteroidetes, Bacteroides, Eubacterium, Alistipes*	(Qin et al., 2014)
HCV-induced cirrhosis, ALC, NASH	Controls vs. ALC	Multi-tagged pyrosequencing Stool sample	*Staphylococcaeae, Enterobacteriaceae, Enterococcaceae*	*Lachnospiraceae, Ruminococcaceae, Clostridiales XIV, Veillonellaceae, Porphyromonadaceae*,	(Bajaj et al., 2014)
HCV-induced cirrhosis, ALC	Controls vs. cirrhosis	Multi-tagged pyrosequencing Stool sample	*Enterobacteriaceae*	*Lachonospiraceae, Ruminococcaceae, Blautia*	(Kakiyama et al., 2013)
Liver cirrhosis (hepatic encephalopathy or no hepatic encephalopathy)	Controls vs. cirrhosis	Multi-tagged pyrosequencing Colonic mucosal sample	*Enterococcus, Burkholderia, Proteus*	*Dorea, Subdoligranulum, Incertae Sedis XIV, Blautia, Roseburia, Faecalibacterium*	(Bajaj et al., 2012a)
ALC, HCV-induced cirrhosis, cryptogenic cirrhosis	Controls vs. cirrhosis	Multi-tagged pyrosequencing Stool sample	*Enterobacteriaceae, Alcaligeneceae, Fusobacteriaceae, Lactobacillaceae, Leuconostocaceae*,	*Ruminococcaceae, Lachnospiraceae*	(Bajaj et al., 2012b)
HBV-induced cirrhosis, ALC	Controls vs. cirrhosis	16S rRNA gene sequencing Stool sample	*Proteobacteria, Fusobacteria, Enterobacteriaceae,Veillonellaceae, Streptococcaceae*	*Bacteroidetes, Lachnospiraceae*	(Chen et al., 2011)

In the stool microbiota of patients with liver fibrosis, the proportion of gram-positive bacteria was found to be decreased and the proportion of gram-negative bacteria was increased, suggesting that gram-negative bacteria are associated with the progression of liver fibrosis (De Minicis et al., 2014; Bajaj et al., 2015). At the phylum level, the gut microbiota of patients with liver fibrosis is dominated by Firmicutes and Bacteroidetes, followed by Actinobacteria and Proteobacteria, which are very low in abundance (Qin et al., 2014; Lelouvier et al., 2016). As fibrosis progresses, the abundance of Firmicutes, Verrucomicrobia, and Actinobacteria decreases, and that of Bacteroidetes and Proteobacteria increases (Chen et al., 2011; Bajaj et al., 2012b; Qin et al., 2014; Lelouvier et al., 2016; Loomba et al., 2017; Schwimmer et al., 2019). In liver fibrosis due to nonalcoholic fatty liver disease (NAFLD) in children, Fusobacteria were found to be decreased in patients with fibrosis (F≥2), whereas in other studies, such as those on hepatitis B virus (HBV)-induced cirrhosis, alcoholic liver cirrhosis (ALC), and NAFLD, Fusobacteria were increased (Chen et al., 2011; Qin et al., 2014; Lelouvier et al., 2016; Schwimmer et al., 2019). At the family level, many studies have shown that beneficial bacteria such as *Lachnospiraceae* and *Ruminococcaceae* are decreased significantly in patients with liver fibrosis, whereas potentially pathogenic bacteria such as *Enterobacteriaceae* are increased significantly (Bajaj et al., 2012b; Kakiyama et al., 2013; Bajaj et al., 2014; Bajaj et al., 2015; Lelouvier et al., 2016). Furthermore, a study by Boursier et al. showed that *Bacteroidaceae* abundance is gradually increased and that *Prevotellaceae* and *Erysipelotrichaceae* abundance is decreased in NAFLD patients as fibrosis progresses (Boursier et al., 2016). At the genus level, the abundance of *Lactobacillus*, *Oribacterium*, *Veillonella*, *Streptococcus*, *Clostridium*, *Prevotella*, and *Ruminococcus* is increased in patients with liver fibrosis, whereas that of *Oscillibacter*, *Lactonifactor*, *Akkermansia*, *Enterococcus*, *Eubacterium*, *Alistipes*, and *Prevotella* is decreased (Qin et al., 2014; Boursier et al., 2016; Schwimmer et al., 2019; Dong et al., 2020). The abundance of *Bacteroides* is significantly increased in patients with NAFLD (F≥2) and significantly decreased in those with cirrhosis (Qin et al., 2014; Boursier et al., 2016). At the species level, they showed increased abundances of *Prevotella copri*, *Bacteroides vulgatus*, and *Escherichia coli* and decreased abundances of *Ruminococcus obeum CAG: 39*, *R. obeum*, and *Eubacterium rectale* (Loomba et al., 2017; Dong et al., 2020).

In addition to studies on the stool microbiota of patients with liver fibrosis, Bajaj et al. studied salivary microbiota and sigmoid mucosa microbiota of patients with cirrhosis and found that both were different from the stool microbiota (Bajaj et al., 2012a; Bajaj et al., 2015). Compared to that in controls, the ratio of salivary microbiota (*Lachnospiraceae + Ruminococcaceae + Clostridiales Incertae Sedis XIV/Streptococcaceae*) was found to be significantly lower in cirrhotic patients. The abundance of *Dorea*, *Subdoligranulum*, *Incertae Sedis XIV*, *Blautia*, *Roseburia*, and *Faecalibacterium* is significantly reduced in the mucosa of the sigmoid colon of patients with cirrhosis, whereas that of *Enterococcus*, *Burkholderia*, and *Proteus* is increased (Bajaj et al., 2012a; Bajaj et al., 2015). In addition, a study by Chen et al. found an increase in *Veillonella*, *Megasphaera*, *Dialister*, *Atopobium*, and *Prevotella* and a decrease in *Neisseria, Haemophilus*, and *SR1 genera Incertae Sedis* in the duodenal mucosa of patients with cirrhosis (Chen et al., 2016). In addition, Lelouvier et al. studied the blood microbiota of patients with liver fibrosis and found that it mainly comprised Proteobacteria and Actinobacteria and that the abundance of Proteobacteria, *Sphingomonas*, and *Bosea* was increased, whereas that of Actinobacteria and *Variovorax* was decreased, in patients with liver fibrosis in comparison to that in controls (Lelouvier et al., 2016).

### 2.2 Effect of altered gut microbiota on liver fibrosis

Although numerous studies have revealed changes in the gut microbiota of patients with liver fibrosis, the mechanisms through which alterations in the gut microbiota affect liver fibrosis are unclear. Current research suggests that the gut microbiota affects liver fibrosis progression primarily by maintaining the intestinal barrier function and regulating metabolic functions and the immune system (Zhou et al., 2019). Intestinal barrier dysfunction and increased intestinal permeability provide channels for the entry of gut microbiota and their metabolites into the liver. Gut microbiota and their metabolites act as messengers that activate inflammatory signaling-related pathways after entering the liver *via* the intestinal barrier, regulating liver fibrosis processes (Zhou et al., 2019; Qi et al., 2020).

The intestinal barrier is the first barrier through which gut lumen contents can enter the liver and is mainly composed of the gut microbiota, an intestinal mucus layer, and intestinal epithelial cells (Camilleri et al., 2012; Chopyk and Grakoui, 2020). The integrity of the intestinal barrier is important to block harmful substances from the intestinal lumen from entering the liver through the portal vein (Schnabl and Brenner, 2014). Dysbiosis of the gut microbiota, an important component of the intestinal barrier, can lead to disruption of the intestinal barrier and increased intestinal permeability (Schnabl and Brenner, 2014; Chopyk and Grakoui, 2020). Gut microbiota can affect intestinal barrier functions *via* a variety of mechanisms (Chopyk and Grakoui, 2020). Disturbed gut microbiota can impair the intestinal barrier function by disrupting the tight intercellular junctions among intestinal epithelial cells, causing an intestinal inflammatory response, inhibiting mucin production, reducing the release of intestinal antimicrobial peptides, and promoting the growth of pathogenic bacteria (Wells et al., 2017; Hiippala et al., 2018; Chopyk and Grakoui, 2020; Paone and Cani, 2020; Gou et al., 2022). Correcting this imbalanced gut microbiota can result in intestinal barrier repair, inhibiting the development of liver fibrosis. For example, ursolic acid and chlorophyll can improve gut microbiota imbalances, repair intestinal barrier functions, and reduce liver fibrosis (Zheng et al., 2018; Wan et al., 2019).

Disruption of the intestinal barrier allows the gut microbiota in the intestinal lumen and its various metabolites, such as bile acids, lipopolysaccharides (LPS), endogenous ethanol, and short-chain fatty acids, to enter the body in large quantities, which plays an important role in the process of liver fibrosis (Ohtani and Kawada, 2019). Bile acids are important gut microbiota metabolites that can disrupt the intestinal barrier and also act as signaling molecules that regulate liver fibrosis *via* a variety of pathways (Fiorucci et al., 2021; Yang et al., 2021). LPS, a major component of the outer membrane of gram-negative bacteria, is recognized by the immune system when it reaches the liver *via* gaps in the intestinal barrier, which in turn activates an inflammatory cascade to exacerbate liver fibrosis (Suk and Kim, 2019; Wan et al., 2019; Lee and Suk, 2020). Trimethylamine N-oxide (TMAO) is derived from the conversion of choline by *Desulfovibrio desulfuricans* and *Escherichia coli*, among others. The increased synthesis of TMAO results in a lack of choline in the body, which in turn enhances oxidative stress in hepatocytes and increases liver inflammation and fibrosis (Schnabl and Brenner, 2014; Giuffrè et al., 2020; He et al., 2021; Jennison and Byrne, 2021). Endogenous ethanol is derived from the fermentation of carbohydrates by bacteria such as Proteobacteria (especially *E. coli* and *Klebsiella pneumonia*). Endogenous ethanol can disrupt the tight junctions between intestinal epithelial cells and cause liver damage similar to that caused by exogenous alcohol, among other substances (He et al., 2021; Jennison and Byrne, 2021; Wu et al., 2021a). Short-chain fatty acids (acetate, propionate, and butyrate) are beneficial metabolites produced *via* the intestinal fermentation of polysaccharides, and these promote liver health *via* the maintenance of intestinal barrier functions and immune homeostasis (Furusawa et al., 2013; Koh et al., 2016; Rau et al., 2018; He et al., 2021). The decrease in short-chain fatty acid-producing flora, including *Akkermansia muciniphila*, *Ruminococcus*, *Faecalibacterium*, and *Eubacterium*, leads to disruption of the intestinal barrier and immune disorders, thereby exacerbating liver fibrosis (Morrison and Preston, 2016; Feng et al., 2018).

The liver is a central immune organ. The gut microbiota and associated metabolites reach the liver *via* the intestinal barrier gaps with portal blood flow, and these are involved in the liver fibrosis process *via* Toll-like receptors (TLRs), nucleotide-binding oligomerization domain-like receptor (NLR)-mediated innate immunity, and T cell receptor immune repertoire (TCR IR)-mediated adaptive immune pathways involved in liver fibrosis (Seki and Schnabl, 2012; Mridha et al., 2017; Liang et al., 2020). The TLR family are transmembrane proteins on intrahepatic cells that activate the innate immune system by recognizing microbiota and metabolites from the intestine, of which, TLR2, TLR4, TLR5, TLR7, and TLR9 can be involved in liver fibrosis, with TLR4 currently being the most studied (Seki et al., 2007; Seki and Schnabl, 2012; Chen et al., 2019a; Lee and Suk, 2020). The transfer of gut microbiota metabolites, such as LPS secondary to intestinal barrier damage, to the liver results in binding to TLR4 on hepatic blastocytes and hepatic stellate cells, activating the TLR4-MyD88-NF-κB signaling pathway, upregulating levels of inflammatory factors such as TNF-α, interleukin IL-1β, and IL-6, stimulating extracellular matrix synthesis by hepatic stellate cells, and causing or exacerbating liver fibrosis (Seki et al., 2007; Bastian et al., 2019; Chen et al., 2019a; Giuffrè et al., 2020). In addition, TLR4 stimulation also promotes liver fibrosis by downregulating the expression of Bambi, an endogenous decoy receptor for the TGF-β receptor, and upregulating the TGFβ/Smad signaling pathway (Seki and Schnabl, 2012; Zhang et al., 2017). In addition to TLR, NLR receptor-mediated innate immunity has an important role in the process of liver fibrosis. NLR-like receptors are a class of pattern recognition receptors expressed mainly in the cytoplasm of liver cells that, upon stimulation, induce the activity of NOD-like receptor protein 3 (NLRP3) inflammatory vesicles in the cytoplasm (Wree et al., 2014). NLRP3 inflammatory vesicles recognize signaling molecules released from injured cells and pathogenic organisms and, *via* the innate immune pathway, mediate caspase-1 activation and produce the cytokines IL-1β and IL-18, which stimulate hepatic stellate cells to promote fibrosis progression (Watanabe et al., 2009; Wree et al., 2014; Chen et al., 2019a). In addition, adaptive immune pathways also play an important role in the progression of liver fibrosis (Koyama and Brenner, 2017). Adaptive immune cells such as T cells and B cells have been found to regulate inflammation and liver fibrosis (Novobrantseva et al., 2005; Hammerich et al., 2014). The intestinal antigens produced by gut microbiota and other organisms enter the portal circulation and are captured by intrahepatic antigen-presenting cells, which activate the adaptive immune system after binding to T-cell receptors (Liang et al., 2020). Dysbiosis of the gut microbiota can promote liver fibrosis by regulating the activation of hepatic stellate cells *via* the TCR IR-mediated intrahepatic immune environment (Liang et al., 2020). Restoring the normal gut microbiota *via* fecal transplantation can reduce liver fibrosis *via* remodeling of the intrahepatic TCR IR, a reduction in B cells, and an increase in CD8+ T cells (Novobrantseva et al., 2005; Guidotti et al., 2015; Liang et al., 2020). In addition, a study by Xu et al. found that *Schistosoma japonicum*-induced liver fibrosis could be alleviated by suppressing the helper T cell 2 immune response and improving gut microbiota dysbiosis (Xu et al., 2020).

## 3 Bile acids and liver fibrosis

The gut microbiota produces numerous metabolites to regulate the host’s metabolism, among which bile acids are very important (Hou et al., 2021; Gu et al., 2022). Bile acids are amphiphilic hydroxysteroids that play a critical role in glucolipid metabolism, energy expenditure, inflammatory response, and signaling regulation in the body (de Aguiar Vallim et al., 2013). Many studies have demonstrated that bile acid metabolism becomes disrupted in patients with liver fibrosis and that this disruption impacts liver fibrosis progression (Caussy et al., 2019; Liu et al., 2019b; Adams et al., 2020; Chen et al., 2020a; Wang et al., 2020; Nimer et al., 2021; Xie et al., 2021).

### 3.1 Enterohepatic circulation of bile acids

**Figure 1:**
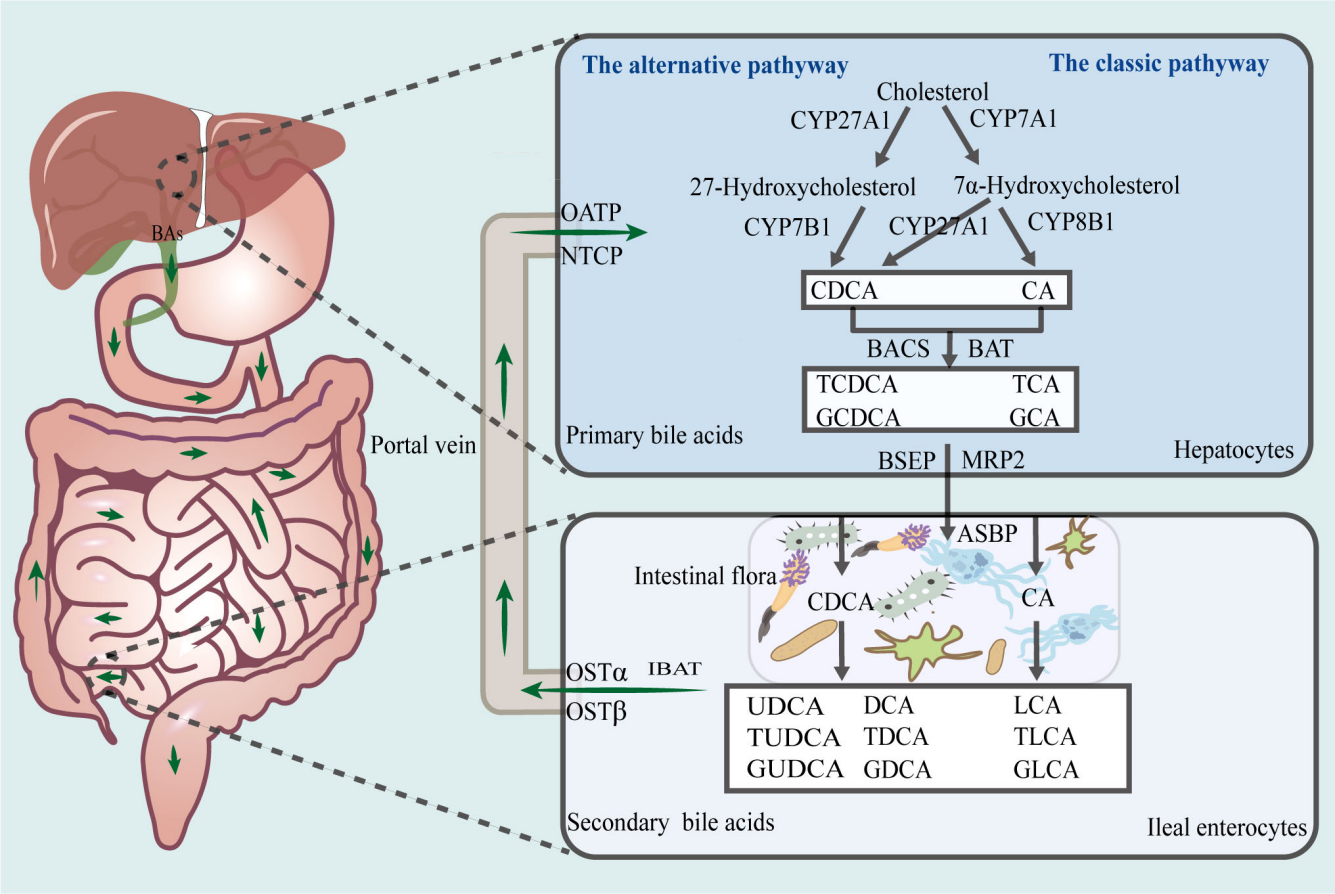
Enterohepatic circulation of bile acids. The liver is the sole site for bile acid synthesis, and there are two main pathways in this regard, namely, the classical and alternative bile acid synthesis pathways. *Via* the action of a series of catalytic enzymes, cholesterol is converted to primary bile acids, CA, TCA, GCA, CDCA, TCDCA, and GCDCA, which thereafter, are transported *via* the biliary tract to the intestines, where they are converted by gut microbiota to secondary bile acids, LCA, TLCA, GLCA, DCA, TDCA, GDCA, UDCA, TUDCA, and GUDCA. Approximately 95% of bile acids are reabsorbed into the liver *via* the enterohepatic cycle. CA, cholic acid; CDCA, chenodeoxycholic acid; TCA, taurocholic acid; GCA, glycocholic acid; TCDCA, taurochenodeoxycholic acid; GCDCA, glycochenodeoxycholic acid; LCA, lithocholic acid; DCA, deoxycholic acid; TLCA, taurolithocholic acid; GLCA, glycolithocholic acid; TDCA, taurodeoxycholate; GDCA, glycodeoxycholic acid; UDCA, ursodeoxycholic acid; TUDCA, tauroursodeoxycholic acid; GUDCA, glycoursodeoxycholic acid.

The liver is the sole site of bile acid synthesis *via* both the classical and alternative pathways, both of which use intrahepatic cholesterol as the initial substrate and involve several enzymatic oxidation processes (**Figure 1**). The rate-limiting enzyme, cholesterol 7-hydroxylase (CYP7A1), initiates the classical pathway, which is the primary mechanism for bile acid synthesis. This process generates 7α-hydroxysteroids, which are later converted by sterol 12α-hydroxylase (CYP8B1) and other enzymes to cholic acid (CA) and a small fraction of chenodeoxycholic acid (CDCA). Additionally, rate-limiting enzymes, such as sterol 27-hydroxylase and oxysterol 7-hydroxylase, participate in the alternative pathway, to synthesize most of the primary bile acid, CDCA (Jia et al., 2018). It has also been observed that the ratio of CA to CDCA among primary bile acids is determined by CYP8B1 activity (Wahlstrom et al., 2016).

Furthermore, CA and CDCA, which are activated by bile acid-CoA synthase and bile acid-CoA amino acid N-acetyltransferase, bind to taurine or glycine to form primary conjugated bile acids, such as taurocholic acid (TCA) and glycocholic acid (GCA). The bile salt export pump and multidrug resistance-associated protein 2 actively transports primary bile acids to the bile duct, after which they are stored in the gallbladder. When they are excreted into the gut after a meal or owing to another stimulus, a small proportion of primary bile acids is transformed by the gut microbiota into secondary free bile acids, such as deoxycholic acid (DCA), lithocholic acid (LCA), and ursodeoxycholic acid (UDCA). Additionally, most primary bile acids are transported to the basolateral membrane of the ileum by the apical sodium-dependent bile acid transporter protein and ileal bile acid transporter protein. Thereafter, they enter the portal circulation owing to the action of the organic solute transporter α/β (OSTα/OSTβ) heterodimer. The Na+/taurocholate cotransporting polypeptide (NTCP) and organic anion transporter protein transport free and conjugated bile acids, respectively, to the liver. Approximately 95% of bile acids in the gut are reabsorbed into the liver *via* the enterohepatic cycle at the end of the ileum, while the remaining unabsorbed portion is excreted with feces and urine. This lost portion of bile acids is replaced *via* hepatic synthesis using raw materials (Hofmann, 2009; Alberto González-Regueiro et al., 2017; Fiorucci et al., 2021; Guzior and Quinn, 2021).

### 3.2 Changes in the bile acid profile of patients with liver fibrosis

Current studies on bile acid metabolism in patients with liver fibrosis mainly focus on serum/plasma and fecal bile acid profiles, with fewer studies examining bile acid metabolism profiles in liver tissues of patients with liver fibrosis, which might be due to the difficulty of obtaining tissue samples in this population (**Table 2**). Numerous studies have demonstrated that serum/plasma total bile acid levels are significantly elevated in patients with liver fibrosis and that total bile acid concentrations are significantly and positively correlated with the degree of liver fibrosis (Caussy et al., 2019; Liu et al., 2019b; Adams et al., 2020; Chen et al., 2020a; Wang et al., 2020; Nimer et al., 2021; Xie et al., 2021). Serum total bile acid levels, in combination with various laboratory indicators, can predict the extent of liver fibrosis with a high degree of accuracy (Shlomai et al., 2013; Yan et al., 2020). A large part of the increase in total serum bile acid levels is due to an increase in primary bile acid levels, especially primary conjugated bile acids, and a decrease in secondary conjugated bile acid levels (Puri et al., 2018; Caussy et al., 2019; Chen et al., 2019b; Liu et al., 2019b; Adams et al., 2020; Chen et al., 2020a; Nimer et al., 2021; Sang et al., 2021). Specifically, among serum/plasma primary bile acids, the levels of glycochenodeoxycholic acid (GCDCA), taurochenodeoxycholic acid (TCDCA), GCA, and TCA are elevated, and the levels of CA, CDCA and muricholate (MCA) are relatively decreased (Puri et al., 2018; Caussy et al., 2019; Chen et al., 2019b; Wang et al., 2020; Xie et al., 2020). In serum/plasma secondary bile acids, the levels of UDCA, tauroursodeoxycholic acid (TUDCA), glycoursodeoxycholic acid (GUDCA), glycochenodeoxycholic acid (GDCA), 7-ketodeoxycholic acid (7-Keto-DCA), Glyco-λ-muricholate (Gλ-MCA), glycolithocholic acid (GLCA), taurodeoxycholate (TDCA), Tauro-λ-muricholate (T λ-MCA), and taurolithocholic acid (TLCA) were elevated (Wang et al., 2016; Liu et al., 2018; Liu et al., 2019b; Adams et al., 2020; Chen et al., 2020a; Nimer et al., 2021). Furthermore, in patients with liver fibrosis, fecal bile acid levels are decreased, fecal primary conjugated bile acids are increased, and secondary bile acids, especially secondary non-conjugated bile acids, such as LCA and DCA, are decreased (Kakiyama et al., 2013; Wang et al., 2020). However, Adams et al. reached the opposite conclusion in their study of fibrosis triggered by NAFLD (Adams et al., 2020). In addition, some differences are observed in the serum bile acid profiles of patients with liver fibrosis triggered by different etiologies. For instance, compared to HBV-induced cirrhosis and non-alcoholic steatohepatitis (NASH), patients with ALC and primary biliary cirrhosis (PBC) had higher levels of total bile acids, TCA, LCA, TCDCA, TUDCA, GUDCA, and conjugated bile acids along with a higher conjugated/unconjugated bile acid and CA/CDCA ratios, while 12-ketoLCA, CDCA, nor-DCA, unconjugated bile acids, total DCA, and conjugated DCA were decreased (Sang et al., 2021) (**Table 2**).

**Table 2:** Changes in the bile acid profile of patients with liver fibrosis.

Causes of fibrosis/type of liver disease	Groups compared	Sample	Results	References
NAFLD	Fibrosis (F0 vs. F1 vs. F2 vs. F3 vs. F4)	Plasma	Increased: total bile acids, primary bile acids (mainly GCDCA and GCA), and 7-Keto-DCA and GUDCA in secondary bile acids.	(Nimer et al., 2021)
NAFLD	Controls vs. F0-1 fibrosis vs. F ≥ 2 fibrosis	Serum	Increased: total bile acids, the ratio of primary conjugated bile acids to secondary conjugated bile acids, the ratio of primary conjugated bile acids to primary bile acids, GCA and GDCA.	(Adams et al., 2020)
		Stool	Increased: total bile acids, secondary unconjugated bile acids, especially DCA and LCA.	
NAFLD	Fibrosis (F0 vs. F1 vs. F2 vs. F3 vs. F4)	Serum	Increased: total bile acids, primary conjugated bile acids Reduced: unconjugated bile acids, especially CA and CDCA	(Caussy et al., 2019)
NASH	Fibrosis (F0 vs. F1 vs. F2 vs. F3 vs. F4)	Serum, liver	Increased: primary bile acids, especially TCDCA and GCDCA, the ratio of conjugated CDCA (TCDCA + GCDCA) to MCA Reduced: secondary bile acid levels, especially MCA	(Chen et al., 2019b)
NASH	F0-1 fibrosis vs. F ≥ 2 fibrosis	Plasma	Increased: TCA and GCA Reduced: the ratio of secondary bile acids to primary bile acids	(Puri et al., 2018)
NASH	Controls vs. fibrosis	Serum	Increased: total bile acids, 12α-OH bile acids (CA, DCA, and their taurine- and glycine-conjugated derivatives)	(Xie et al., 2021)
HBV-induced fibrosis	stages 0–2 fibrosis vs. stages 3–4 fibrosis	Serum	Increased: TCA, combined with Tyr/Val ratio, Tyr, age and other indicators can predict advanced fibrosis	(Xie et al., 2020)
HBV-induced fibrosis	Controls vs. F0-1 fibrosis vs. F2-4 fibrosis	Serum	Increased: total bile acids, primary bile acids, primary conjugated bile acids, TCDCA, GCDCA, GCA, and TCA	(Wang et al., 2020)
		Stool	Increased: primary conjugated bile acids Reduced: total bile acids, unconjugated bile acids, secondary bile acids especially unconjugated secondary bile acids, LCA, and DCA	
HBV-induced fibrosis	Fibrosis (F0 vs. F1 vs. F2 vs. F3 vs. F4)	Serum	Increased: total bile acids, and the ratio of total bile acids to total cholesterol was one of the independent predictors of significant fibrosis.	(Yan et al., 2020)
HCV-induced fibrosis	F0-F2 fibrosis vs. F3-F4 fibrosis	Serum	Increased: total bile acids, combined with a broad range of laboratory parameters can predict the degree of fibrosis with high accuracy	(Shlomai et al., 2013)
HBV-induced cirrhosis	Controls vs. cirrhosis	Serum	Increased: GCDCA, GCA, TCA, TCDCA, GDCA, GUDCA, Gλ-MCA, GLCA, CDCA, CA, UDCA, λ-MCA, TUDCA, TDCA, T λ-MCA and TLCA	(Wang et al., 2016)
Viral hepatitis-induced cirrhosis, ALC, autoimmune cirrhosis	Early stage cirrhosis vs. middle stage cirrhosis vs. late stage cirrhosis	Serum	Increased: total bile acids, primary conjugated bile acids (GCA, GCDCA, TCDCA, and TCA), TUDCA	(Liu et al., 2019b)
HCV-induced cirrhosis, ALC, hepatic encephalopathy	Controls vs. early cirrhosis vs. advanced cirrhosis	Serum	Increased: conjugated bile acids, unconjugated secondary bile acids	(Kakiyama et al., 2013)
		Stool	Reduced: total bile acids, secondary bile acids, the ratio of secondary bile acids to primary bile acids, especially DCA/CA and LCA/CDCA	
ALC, PBC, HBV-induced cirrhosis, NASH	(HBV-induced cirrhosis, NASH) to (ALC, PBC)	Serum	Increased: total bile acids, TCA, LCA, TCDCA, TUDCA, GUDCA, conjugated bile acids, conjugated/unconjugated, CA/CDCA ratio Reduced: 12-ketoLCA, CDCA, Nor-DCA, Unconjugated BAs, total DCA, and conjugated DCAs	(Sang et al., 2021)
HBV-induced cirrhosis, ALC, PBC, cryptogenic cirrhosis	Controls vs. cirrhosis	Serum	Increased: primary bile acids, TCA, TCDCA, TUDCA, GCA, UDCA, CDCA, CA, TLCA, TDCA, HDCA, and LCA	(Liu et al., 2018)
PBC	Controls vs. PBC	Serum	Increased: total bile acids, primary bile acids, GCA, TCA, GCDCA Reduced: secondary bile acids, DCA, LCA, TLCA, TDCA, GDCA	(Chen et al., 2020a)

### 3.3 Role of bile acids in liver fibrosis progression

**Figure 2:**
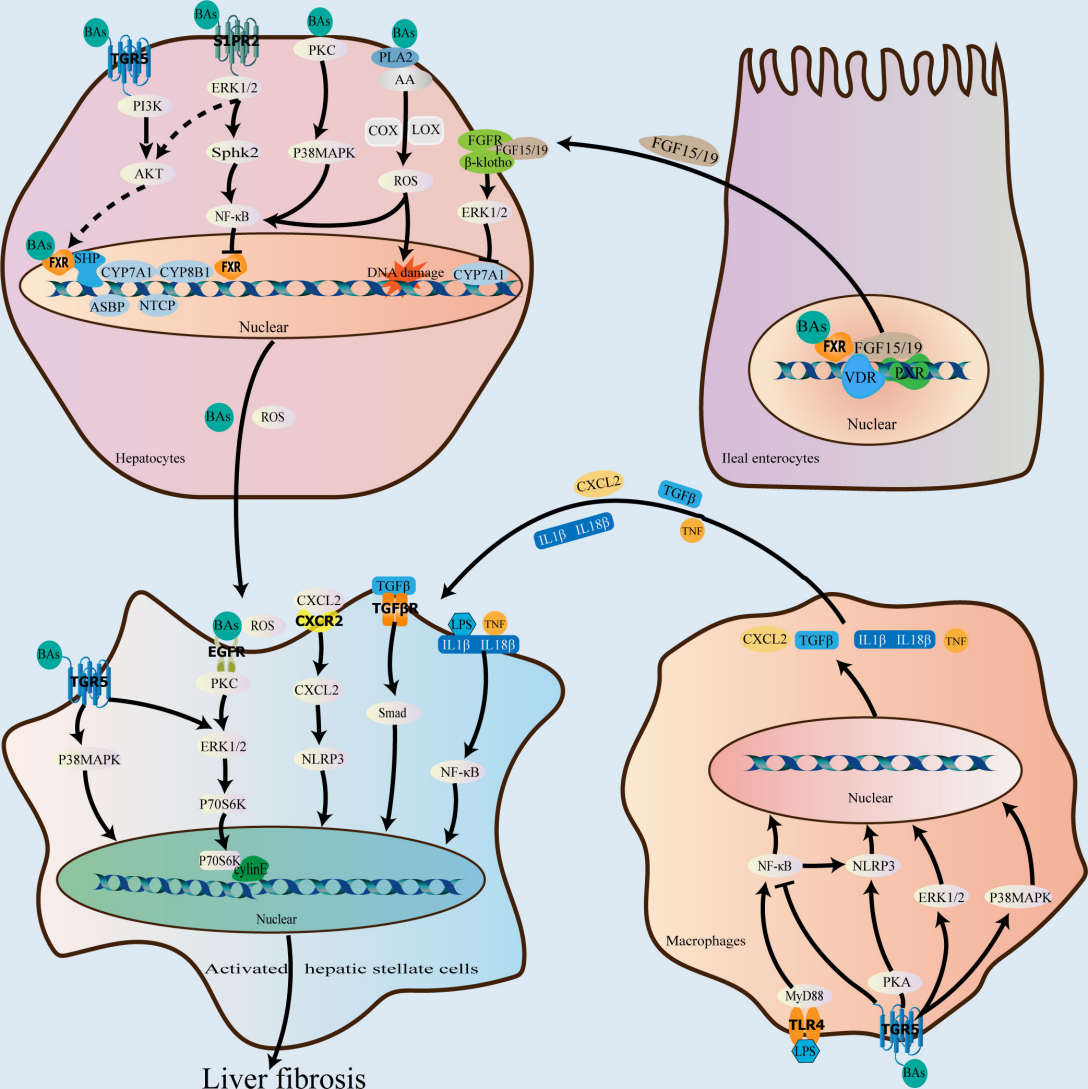
Role of bile acids in the progression of liver fibrosis. As important signaling molecules, bile acids can participate in liver fibrosis progression by binding to receptors in hepatocytes, macrophages, ileal enterocytes, and hepatic stellate cells. In the liver, bile acids bind to FXR and downregulate CYP7A1, CYP8B1, NTCP, and ASBP, and upregulate BSEP and OSTα/β *via* the FXR-SHP signaling pathway to the end of attenuating bile acid synthesis and promoting their excretion, thereby preventing and attenuating liver fibrosis caused by bile acid accumulation. In the ileum, bile acids bind to FXR to downregulate CYP7A1, CYP7B1, and CYP8B1 *via* the FXR-FGF19/15 signaling pathway; VDR and PXR also play a role in regulating FGF19/15expression. After bile acids bind to the membrane receptor, TGR5, in addition to coordinating the effect of FXR, they can also activate the p38 MAPK and ERK1/2 pathways and inhibit the NF-κB pro-inflammatory signaling pathway to exert an antifibrotic effect. In contrast, bile acid binding to S1PR2 and EGFR can promote the occurrence of liver fibrosis *via* the ERK1/2 signaling pathway. Additionally, bile acids can directly damage the plasma membrane to activate the p38 MAPK, NF-κB, and PLA2 signaling pathways, resulting in an increase in reactive oxygen species levels in hepatocytes and the promotion of liver fibrosis. FXR, farnesoid X receptor; VDR, vitamin D receptor; SHP, small heterodimer partner; CYP7A1, cholesterol 7α-hydroxylase; CYP8B1, 12α-hydroxylase; NTCP, Na^+^/taurocholate cotransporting polypeptide; ABSP, apical sodium-dependent bile acid transporter protein; BSEP, bile salt export pump; OSTα/β, organic solute transporter α/β; CYP7B1, oxysterol 7α-hydroxylase; FGF19/15, fibroblast growth factor 19/15; PXR, pregnane X receptor; TGR5, G protein-coupled receptor 5; ERK, extracellular signal-regulated kinase; PLA2, phospholipase A2; S1PR2, sphingosine 1-phosphate receptor 2; MAPK, mitogen-activated protein kinase; NF-κB, nuclear factor kappa B; EGFR, epidermal growth factor receptor.

Bile acids function as important signaling molecules that activate nuclear receptors, such as farnesoid X receptor (FXR), pregnane X receptor (PXR), and vitamin D receptor (VDR), as well as membrane receptors, including G protein-coupled receptor 5 (TGR5) and sphingosine 1-phosphate receptor 2 (S1PR2), to maintain the homeostasis of the bile acid enterohepatic cycle. They are also involved in the process of liver fibrosis progression (Fiorucci et al., 2021) (**Figure 2**).

Specifically, the activation of FXR by bile acids decreases bile acid production and enhances bile acid excretion *via* a negative feedback effect, preventing and reducing liver fibrosis progression induced by excessive bile acid accumulation (Pollheimer et al., 2014). The primary bile acid, CDCA, is the most effective ligand for FXR, which is extensively expressed in the liver and intestines, and the order of the binding efficacy of common bile acids as ligands with respect to FXR activation, from strong to weak, is: CDCA>CA>LCA>deoxycholic acid. Additionally, bile acid-mediated FXR activation in the liver induces the expression of small heterodimer partner (SHP), which is associated with the nuclear orphan receptors, liver receptor homologue 1 and hepatocyte nuclear factor 4α binding, and activates the FXR-SHP signaling pathway, downregulates *CYP7A1* and *CYP8B1* expression, and regulates bile acid synthesis *via* a negative feedback mechanism (Fiorucci et al., 2021). Hepatic FXR also reduces bile acid uptake and promotes bile acid excretion by downregulating Na^+^/taurocholate cotransporting polypeptide and the apical sodium-dependent bile acid transporter protein *via* SHP-dependent processes and by upregulating bile salt export pump and OSTα/β (Sinal et al., 2000; Hwang et al., 2002). After activation by bile acids, ileal FXR mediates fibroblast growth factor 19/15 expression by binding to the fibroblast growth factor receptor 4/β- klotho complex. This triggers the extracellular signal-regulated kinase (ERK)1/2 and c-Jun N-terminal kinases (JNK) signaling pathways in hepatocytes, thereby downregulating *CYP7A1*, oxysterol 7α-hydroxylase, and *CYP8B1*, and attenuating bile acid production (Inagaki et al., 2005; Kong et al., 2012). In addition to FXR, ileal FGF19/15 expression is regulated by various receptors, including VDR and PXR, which inhibit *CYP7A1* expression by competing for the binding of HNF4α to BARE-II (Schmidt et al., 2010; Fiorucci et al., 2021).

Additionally, bile acids exert antifibrotic effects in combination with TGR5, which is widely expressed in the liver, spleen, kidneys, intestines, and gallbladder tissues as well as macrophages, with the secondary bile acid, LCA being its most potent ligand. Further, the order of the binding efficacy of common bile acids with respect to TGR5 activation, from strong to weak is: LCA>DCA>CDCA>CA. Specifically, TGR5 partially overlaps with FXR in function and may play a synergistic role in FXR-mediated regulation of bile acid production (Fiorucci et al., 2021). The binding of bile acids to TGR5 can exert anti-inflammatory and antifibrotic effects *via* the activation of the cyclic phosphatidyl adenosine/protein kinase A signaling pathway, inducing the phosphorylation of NLR family pyrin domain containing 3 inflammatory vesicles (Guo et al., 2016). Additionally, TGR5 activation by bile acids inhibits the lipopolysaccharide-induced NF-κB pro-inflammatory signaling pathway in hepatic Kupffer cells and also exerts anti-inflammatory effects (Chiang and Ferrell, 2020). It has also been observed that TGR5 activation by bile acids triggers the p38 mitogen-activated protein kinase (p38 MAPK) signaling pathway, which activates the ERK1/2 and JNK pathways, thereby exerting anti-inflammatory and antifibrotic effects (Keitel et al., 2019).

S1PR2 is a G protein-coupled receptor for which bound bile acids are potent ligands. In hepatocytes, TCA binds to S1PR2 and activates the ERK1/2 and AKT pathways (Studer et al., 2012). Particularly, the AKT pathway plays an important role in mediating bile acid binding to FXR (Cao et al., 2010), and S1PR2 functioning in combination with FXR may facilitate bile acid pool regulation. Additionally, TCA may promote liver fibrosis by binding to S1PR2 and activating the ERK1/2-sphingosine kinase 2 signaling pathway *via* the upregulation of genes involved in cell proliferation and metabolism and the induction of NF-κB expression. Conversely, the knockdown of *S1pr2* in mice reduces liver fibrosis induced by bile duct ligation (Keitel et al., 2019; Hou et al., 2020).

Svegliati-Baroni et al. observed that GCDCA activates the epidermal growth factor receptor, inducing hepatic stellate cell proliferation and liver fibrosis (Svegliati-Baroni et al., 2005), and this may be related to the activation of the epidermal growth factor receptor/MEK/ERK signaling pathway (Hohenester et al., 2020). Zhang et al. also showed that GCDCA may promote liver fibrosis by regulating the p38/JNK signaling pathway to promote the proliferation and motility of hepatic stellate cells (Zhang et al., 2010). Additionally, bile acids can damage the plasma membrane directly, causing PKC activation, which activates the p38 MAPK pathway as well as the NF-B signaling pathway while promoting liver fibrosis. Membrane disruption by bile acids also activates cytoplasmic phospholipase A2, which releases arachidonic acid from the membrane *via* cyclooxygenase and lipoxygenase activity, ultimately resulting in an increase in the level of reactive oxygen species in hepatocytes and a pro-fibrotic impact (Jia et al., 2018).

## 4 The gut microbiota–bile acid axis in liver fibrosis

### 4.1 The gut microbiota–bile acid axis

The interaction between the gut microbiota and bile acids is complex and bidirectional in the host, known as the gut microbiota–bile acid axis (Hou et al., 2021; Wu et al., 2021b; Jiang et al., 2022). The gut microbiota can transform and modify bile acids, influence bile acid production, and alter bile acid receptor signaling. In turn, bile acids can effectively regulate the growth of the gut microbiota and maintain intestinal homeostasis (Ramírez-Pérez et al., 2017; Li et al., 2021).

Numerous studies have confirmed the correlation between the gut microbiota and bile acids in different diseases. In inflammatory bowel disease, the primary bile acids CA, CDCA, and their conjugates were found to be positively correlated with the abundance of *Enterococcus*, *Ruminococcus gnavus*, *Clostridium clostridioforme*, *Klebsiella*, and *Streptococcus*, whereas they were determined to be negatively correlated with the abundance of *Alistipes finegoldii* and other taxa. Secondary bile acids such as DCA and LCA are positively correlated with the abundance of members of the Firmicutes such as *Clostridiales, Enterococcaceae, Eubacteriaceae, Lachnospiraceae*, and *Ruminococcaceae* in the gut and negatively correlated with the abundance of *Enterococcus*, *Lactobacillus*, and others (Connors et al., 2020; Li et al., 2021; Yang et al., 2021). Jiang et al. found that *Ruminococcaceae UCG-002* and *UCG-003* are positively correlated with secondary bile acids (IsoLCA, LCA and UDCA) and negatively correlated with primary bile acids (MCA and NorCA) in chronic insomnia and cardiometabolic disease (Jiang et al., 2022). In addition, Wei et al., in a study of obesity susceptibility, found that *Clostridium scindens* is positively correlated with LCA, DCA, and UDCA and that *Clostridium hylemonae* is significantly positively correlated with UDCA (Wei et al., 2020). In addition, alterations in the bile acid axis of the gut microbiota have been found to be associated with the development of gastrointestinal inflammation, metabolism-related fatty liver disease, liver fibrosis, liver cancer, and colon cancer (Jia et al., 2018; Ma et al., 2018; Qi et al., 2019; Wu et al., 2021b).

#### 4.1.1 Role of gut microbiota in bile acid metabolism

The gut microbiota can influence the synthesis of and metabolic processes associated with bile acids *via* a variety of mechanisms (Guzior and Quinn, 2021). In the intestine, primary bile acids are biotransformed into secondary bile acids by enzymes produced by the gut microbiota *via* deconjugation, dehydroxylation, oxidation and isomerization, esterification and desulfation reactions (Pavlidis et al., 2015; Thomas et al., 2022). Bacteria with bile salt hydrolase activity (*Bacteroides, Clostridium, Lactobacillus, Bifidobacterium*, *Enterococcus, Ruminococcaceae*, and *Listeria*) dominate the bile acid deconjugation process (Ridlon et al., 2006; Long et al., 2017; Guzior and Quinn, 2021; Jiang et al., 2022; Thomas et al., 2022). Furthermore, hydroxysteroid dehydrogenases produced by the gut microbiota such as Clostridium are necessary for catalyzing the oxidation, isomerization, and dehydroxylation of bile acids (Guzior and Quinn, 2021). *Clostridium* and *Eubacterium* contribute to bile acid dehydroxylation (Jia et al., 2018); *Bacteroides, Eubacterium, Clostridium, Escherichia, Egghertella, Eubacterium, Peptostreptococcus*, and *Ruminococcus* contribute to their oxidation and isomerization (Jia et al., 2018; Guzior and Quinn, 2021); *Bacteroides, Eubacterium*, and *Lactobacillus* contribute to their esterification (Just et al., 2018); and *Clostridium, Fusobacterium, Peptococcus*, and *Pseudomonas* contribute to their desulfation (Long et al., 2017; Jia et al., 2018).

In addition to their ability to directly convert primary bile acids to secondary bile acids, the gut microbiota can also control bile acid synthesis by regulating bile acid synthesis enzymes. Sayin et al. showed that the gut microbiota could reduce tauro-beta-muricholic acid levels in the intestine and increase hepatic CYP7A1 expression, whereas CYP8B1 activity remains unaffected, thus changing bile acid composition (Sayin et al., 2013). Kwon et al. reported that the expression of bile acid synthesis genes, such as *Cyp7a1*, oxysterol 7α-hydroxylase, *Cyp27a1*, and *Cyp8b1*, is considerably upregulated in the livers of mice fed with *Lactobacillus plantarum*, increasing bile acid synthesis (Kwon et al., 2020). Furthermore, Degirolam et al. observed that the VSL#3 probiotic increases bile acid uncoupling and promotes fecal bile acid excretion, which upregulates the expression of CYP7A1 and CYP8B1 and increases bile acid synthesis (Degirolamo et al., 2014).

#### 4.1.2 Role of bile acids in determining the composition and function of gut microbiota

Bile acids regulate the composition and distribution of gut microbiota. Owing to their amphiphilic structure, bile acids exert cytotoxicity and antibacterial activity. In individual bile acids, a higher hydrophobicity corresponds to a higher virulence and antibacterial activity (de Aguiar Vallim et al., 2013; Baiocchi et al., 2019). In addition to directly affecting damage on bacterial membranes, resulting in DNA damage and protein denaturation (direct antibacterial effects) (Ridlon et al., 2016), bile acids can indirectly mediate the inhibition of microbial growth by regulating the expression of nitric oxide synthase and antimicrobial peptide genes (Gadaleta et al., 2011). Furthermore, they can inhibit bacterial overgrowth in the small intestine, protect intestinal barrier function, and inhibit bacterial translocation (Devkota and Chang, 2015). Lorenzo-Zuniga et al. observed that the oral administration of conjugated bile acids effectively suppresses bacterial overgrowth in the small intestine, bacterial translocation, and endotoxemia in cirrhotic rats (Lorenzo-Zuniga et al., 2003). However, excessive bile acid accumulation can cause intestinal barrier damage and bacterial translocation. Bile duct ligation for seven days can cause bacterial translocation into lymph nodes and for 21 days can expand bacterial translocation to the liver, spleen, lungs, and other organs in rats (Clements et al., 1996).

### 4.2 Gut microbiota–bile acid crosstalk in liver fibrosis

Several studies have revealed that gut microbiota composition and bile acid metabolism are disrupted in liver fibrosis (Kakiyama et al., 2013; Adams et al., 2020; Xie et al., 2021). However, their relationship in liver fibrosis remains unclear. Adams et al. found that the more severe the degree of liver fibrosis in patients with NAFLD, the higher the levels of serum GCA and stool DCA, and that serum GCA and stool DCA levels were positively correlated with *Lachnospiraceae* and negatively correlated with *Bacteroidaceae* (Adams et al., 2020). A study by Lee et al. found that stool primary bile acids in patients with NAFLD were negatively correlated with *Ruminococcaceae* and positively correlated with *Veillonellaceae*. As fibrosis increased, the abundance of *Ruminococcacea* decreased and the abundance of *Veillonellaceae* increased in non-obese NAFLD patients. Moreover, *Ruminococcaceae* and *Veillonellaceae* combined with stool CA, CDCA, UDCA, and propionate) could diagnose non-obese NAFLD patients with fibrosis (Lee et al., 2020). In patients with HBV-induced liver fibrosis, the more severe the fibrosis, the higher the levels of total serum primary bile acids and conjugated primary bile acids and the lower the levels of total stool bile acids and stool unconjugated bile acids. In patients with such fibrosis, Wang et al. found that stool primary bile acids and secondary bile acids were negatively correlated with *Ruminococcus* and *Escherichia*, respectively (Wang et al., 2020). Furthermore, the ratio of the total bile acids to secondary/primary bile acids in stool is reduced, and serum primary bile acid levels are increased in patients with cirrhosis. Additionally, Enterobacteriaceae were positively correlated with CDCA levels, whereas Ruminococcaceae were positively correlated with DCA levels (Kakiyama et al., 2013).

Xie et al. established an animal model of liver fibrosis using three different methods and observed a considerable increase in total bile acid and conjugated secondary 12α-hydroxylated bile acids levels in serum and liver tissues. They also found a positive correlation between gut microbiota and bile acid dissociation, dehydroxylation, and degradation processes and an increase in conjugated secondary 12α-hydroxylated bile acids (Xie et al., 2021). Furthermore, conjugated secondary 12α-hydroxylated bile acids, especially taurodeoxycholic acid and GDCA, promoted liver fibrosis progression by binding to TGR5 and activating hepatic stellate cells *via* ERK1/2 and p38 MAPK signaling (Okubo et al., 2013; Xie et al., 2021). In patients with liver fibrosis, the gut microbiota may alter the intestinal bile acid pool, stimulating the differentiation of naive T cells into Th17 cells, which later migrate into the liver to secrete interleukin-17 and -22. This activates hepatic stellate cells and Kupffer cells, leading to excessive extracellular matrix synthesis and exacerbation of liver fibrosis (Meng et al., 2012; Hang et al., 2019; Chen et al., 2020b).

### 4.3 The gut microbiota–bile acid axis as a target for liver fibrosis treatment

The gut microbiota–bile acid axis can be a potential therapeutic target for delaying or reversing liver fibrosis. In several studies, bile acids or their receptor agonists have shown potential for application in the treatment of liver fibrosis. Specifically, in a rat model of NASH, combining UDCA with losartan attenuated liver fibrosis, decreased intestinal permeability, and inhibited the expression of TGF-β1 and TLR4 with hepatic stellate cells activation (Namisaki et al., 2016). Sombetzki et al. found that 24 nor-UDCA ameliorates the extent of hepatic fibrosis in a mouse model of hepatic schistosomes (Sombetzki et al., 2015). Furthermore, BAR502, a dual agonist of FXR and TGR5, reversed high-fat diet (HFD)- or carbon tetrachloride (CCL4)-induced hepatic fibrosis in mice by upregulating the expression of SHP and FGF15 and attenuating bile acid synthesis (Carino et al., 2017). FXR agonists can also exert antifibrotic effects. In particular, obeticholic acid, which is a semisynthetic derivative of CDCA, reverses liver fibrosis and cirrhosis and has been used to treat patients with primary biliary cirrhosis who fail to respond adequately to ursodeoxycholic acid treatment (Verbeke et al., 2016; Lleo et al., 2020). Fiorucci et al. also showed that when bound to FXR, obeticholic acid attenuates bile acid synthesis and inhibits the proliferation of hepatic stellate cells *via* the FXR-SHP signaling pathway, thereby delaying liver fibrosis progression (Fiorucci et al., 2004). The FXR agonists WAY-362450 and Cilofexor (GS-96740) slowed down liver fibrosis progression (Zhang et al., 2009; Trauner et al., 2019; Schwabl et al., 2021). In addition, TGR5 agonists may exert antifibrotic effects, and TGR5 deficiency exacerbates liver fibrosis in mice (Ferrell et al., 2019). Furthermore, FGF19 and its analogs, NGM282 and M70, inhibited the synthesis of bile acids and significantly improved liver fibrosis (Zhou et al., 2017; Harrison et al., 2020).

As already mentioned above, targeting bile acids is a potential treatment strategy for liver fibrosis. However, it is unclear whether targeting the gut microbiota can reduce liver fibrosis by regulating bile acids. Studies have also shown that several foods or drugs can reduce the progression of liver fibrosis by affecting the gut microbiota and thus altering bile acid metabolism. Additionally, the Western-style high-fat/high-sucrose diet ameliorated liver fibrosis in mice with human-like bile acid composition by inducing changes in gut microbiota composition and regulating bile acid metabolism (Iwamoto et al., 2021). Moreover, exogenous *Lacticaseibacillus rhamnosus* GG supplementation may modulate gut microbiota and thus increase bile acid excretion, thereby ameliorating fibrosis induced by intrahepatic bile acid accumulation in both humans and animals (Liu et al., 2020). Oral antibiotics are also effective in inhibiting intestinal bacterial growth, decreasing portal secondary bile acid levels, and reducing liver inflammation and fibrosis (Janssen et al., 2017). Si-Wu-Tang ameliorated liver fibrosis by regulating gut microbiota composition to reduce pathogenic bile acid levels and enhance the synthesis of beneficial bile acids (Xue et al., 2021). Furthermore, Yin-Chen-Hao Tang attenuated CCL4-induced liver injury by modulating the gut microbiota to reduce the levels of metabolites, such as TCA (Liu et al., 2019a). Nicotinamide riboside also improved alcoholic liver injury by regulating the gut microbiota–bile acid axis (Yu et al., 2021).

## 5 Conclusion

Liver fibrosis, a common stage in various chronic liver injuries, is a serious condition that considerably affects human health. Thus, its treatment is a research priority. Studies have shown that the gut microbiota–bile acid axis is closely related to liver fibrosis progression. First, the gut microbiotas of patients with liver fibrosis are significantly altered. This alteration greatly affects liver fibrosis progression. Second, bile acid metabolism is also significantly altered in patients with liver fibrosis, and this dysregulation is also involved in liver fibrosis processes *via* multiple signaling pathways. In addition, bile acids are metabolites of the gut microbiota, and bile salt hydrolases and hydroxysteroid dehydrogenases produced by the gut microbiota are able to convert primary bile acids to secondary bile acids *via* deconjugation, dehydroxylation, desulfation, oxidation, and isomerization. The gut microbiota can also influence bile acid synthesis by affecting synthesis-associated enzymes and pathways. Bile acids, in turn, can affect the abundance and composition of the gut microbiota *via* their own cytotoxic effects and the signaling pathways they mediate. It is well known that bile acids act as messengers between the liver and intestine, which prompted previous studies to suggest that gut microbiota may influence liver fibrosis processes by affecting bile acid metabolism. Therefore, in this review, we examined the alteration of the gut microbiota and bile acid metabolism in patients with liver fibrosis and illustrated the role of the gut microbiota–bile acid axis in liver fibrosis progression, as well as several potential treatments for liver fibrosis that target this axis. We anticipate that future studies will further examine the regulation of the gut microbiota–bile acid axis and potentially pave the way to the delay or even reversal of liver fibrosis.

However, the present studies have some limitations. For example, many clinical studies have limited sample sizes, and the findings are not completely reliable. Multicenter studies with larger sample sizes would be necessary for more credible conclusions. In addition, the existing studies mainly focus on the alteration of the gut microbiota and bile acid metabolism in patients with liver fibrosis, but relatively few studies investigate how gut microbiota and bile acid metabolites affect liver fibrosis. This highlights the need for more studies that aim to reveal the causal relationship between gut microbiota, bile acids, and liver fibrosis and their interrelated mechanisms with the ultimate goal of utilizing the gut microbiota–bile acid axis in clinical practice. Moreover, the methods currently used for gut microbiota detection are mostly 16S rRNA gene sequencing and Metagenomic shotgun sequencing, and the samples used are mostly feces, both of which may not accurately reflect the status of the gut microbiota. Furthermore, most studies on bile acid profile in patients with liver fibrosis have focused on the alteration of serum and fecal bile acid profiles, while studies on bile acid profile in the liver have been less frequent due to the difficulty of obtaining tissue samples. As the liver is the only site of bile acid synthesis, more studies on the bile acid profile of liver tissues are needed to fully reveal the role of bile acids in liver fibrosis in terms of its synthesis, transport, metabolism, and function.

Encouragingly, a large number of studies have now shown that the gut microbiota–bile acid axis is a potential target for liver fibrosis treatment, which might potentially help in treating liver fibrosis, a health problem that has plagued the medical community for years. We believe that with the continuous advancement in research technologies, optimization of research methods, and enhancement of research mechanisms, it might be possible to target the gut microbiota–bile acid axis with the aim of delaying or even reversing liver fibrosis *via* the use of specific foods, antibiotics, probiotics, prebiotics, and bile acid receptor agonists. This will provide specific strategies for the treatment of liver fibrosis and reduce the incidence of cirrhosis and primary liver cancer.

## Author contributions

All authors listed have made a substantial, direct, and intellectual contribution to the work and approved it for publication.

## Funding

This work was supported by the National Natural Science Foundation of China (31960236), the Natural Science Foundation of Gansu Province (21JR7RA369), Lanzhou Talent Innovation and Entrepreneurship Project (2019-RC-34), Lanzhou Chengguan District Science and Technology Planning Project (2020SHFZ0029), and the Fund of the first Hospital of Lanzhou University (ldyyyn2019-75).

## Acknowledgments

We would like to thank Editage (www.editage.cn) for English language editing and Long-Fei Ren and Jia-Hui Xi for critically revising the manuscript.

## Conflict of interest

The authors declare that the research was conducted in the absence of any commercial or financial relationships that could be construed as a potential conflict of interest.

## Publisher’s note

All claims expressed in this article are solely those of the authors and do not necessarily represent those of their affiliated organizations, or those of the publisher, the editors and the reviewers. Any product that may be evaluated in this article, or claim that may be made by its manufacturer, is not guaranteed or endorsed by the publisher.
